# FluvDepoSet: A dataset of synthetic 3D models of fluvial deposits

**DOI:** 10.1016/j.dib.2026.113163

**Published:** 2026-08-13

**Authors:** Guillaume Rongier, Luk Peeters

**Affiliations:** a Department of Geoscience & Engineering, Delft University of Technology, Stevinweg 1, 2628 CN, Delft, The Netherlands; b VITO Digital Water & Soils, Boeretang 200, 2400 Mol, Belgium

**Keywords:** Subsurface, Stratigraphic modeling, Meandering river, Landscape evolution model, Machine learning, Sensitivity analysis

## Abstract

Sediments deposited by rivers constitute a key source for the water, energy, and raw materials that fuel our everyday lives. Finding and managing these resources require to predict the spatial distribution of those fluvial deposits in the subsurface. This remains a major challenge due to the scarcity and poor quality of subsurface data, but also because traditional modeling approaches struggle to reproduce the continuity of those deposits while conditioning those data. To help tackle this challenge, we introduce FluvDepoSet, a large dataset of synthetic 3D models of fluvial deposits. Contrary to common practice, the 20,200 samples in FluvDepoSet were generated using a landscape evolution model, CHILD. CHILD simulates the processes driving landscape evolution, which leads to a high computational cost but ensures that the resulting 3D models are consistent with geological principles. Those processes include river lateral migration, aggradation, and incision with overbank deposition to simulate the evolution of a meandering river over tens of thousands of years. While all the samples share the same basic setting, seven parameters controlling those processes are randomly drawn from uniform distributions for each sample. This results in the buildup of different stratigraphies through time, with coarse sediments deposited in point bars along the river and fine sediments deposited in the floodplain. Those stratigraphies are then transferred to a regular structured grid, which is common to all samples and includes the fraction of coarse sediments and the deposition time. These properties are stored in HDF5 files, each file corresponding to one sample. Thanks to its large number of samples, FluvDepoSet is well-suited for sensitivity analyses to better understand the impact of fluvial deposits on applications – for instance through subsurface flow and transport simulations – and for machine learning to better support subsurface characterization and decision-making – for instance through the generation of more plausible conditional models of fluvial deposits.

Specifications Table

**Table:** 

Subject	Earth & Environmental Sciences
Specific subject area	Stratigraphic modeling of fluvial systems
Type of data	3D image (.h5 format)
Data collection	Data samples were generated using the landscape evolution model CHILD [1] available at https://github.com/grongier/child based on input parameters randomly drawn from pre-defined ranges. For each sample, CHILD's outputs were post-processed using Python and the package pyrunchild available at https://github.com/grongier/pyrunchild to transfer the variables of interest to a regular grid and save it in a HDF5 file.
Data source location	CSIRO, Kensington, WA, Australia
Data accessibility	*Repository name: FluvDepoSet* *Data identification number: 10.25919/4fyq-q291* *Direct URL to data:*https://doi.org/10.25919/4fyq-q291
Related research article	*Rongier, G., & Peeters,* L. *(2022). How fluvial deposits distort aquifer recharge estimated from groundwater age: Insights from a landscape evolution model. Water Resources Research, 58, e2021WR030963.*https://doi.org/10.1029/2021WR030963

## 1 Value of the Data

• Fluvial deposits play a crucial role in our society, either indirectly through the resources they host, for instance groundwater, metals, or heat, or directly through their storage capacity, for instance to store hydrogen or CO_2_ underground. But predicting their distribution remains a major challenge due to the scarcity and poor quality of subsurface data.
• FluvDepoSet provides 20,200 3D synthetic models of fluvial deposits generated by a landscape evolution model, CHILD. Contrary to the object-based approaches often used to generate those deposits, this process-based approach ensures that the spatial distribution of sediments in FluvDepoSet’s models aligns with geological principles.
• Typically, models of fluvial deposits focus on facies, i.e., clusters of rocks with similar characteristics, which is more an interpretation than a measurable quantity. FluvDepoSet focuses instead on variables closer to subsurface data, from which facies can be derived if needed.
• By providing 3D models true to geological principles, FluvDepoSet forms a unique resource to develop and test new algorithms for subsurface characterization and decision-making. By providing an easy-to-use dataset, it can create a bridge between the subsurface and machine learning communities to foster new technical developments.
• Thanks to its large number of samples, FluvDepoSet also forms a valuable resource for sensitivity analyses aiming at better understanding the impact of fluvial heterogeneities on applications such as groundwater contamination, heat production, or hydrogen storage.

## 2 Background

Owing to their permeable nature, sediments deposited by rivers host diverse resources invaluable to our society. But these fluvial deposits are not homogeneous: their properties vary spatially, sometimes drastically and over short distances. Rongier & Peeters [2] showed how these heterogeneities can lead to large errors when estimating groundwater recharge in aquifers. This study is part of a wider corpus demonstrating the importance of considering fluvial heterogeneities when managing subsurface resources, be it groundwater, heat, ore, or storage space for H_2_ or CO_2_ [e.g., [3], [4], [5]].

But subsurface data are too scarce and poorly resolved to capture those heterogeneities, so we need (i) to better understand the impact of heterogeneities on the exploration and management of subsurface resources, and (ii) to develop geological modeling approaches that reproduce the impactful heterogeneities. FluvDepoSet aims at supporting both objectives, with a focus on sensitivity analyses [as illustrated in 2], and the development and test of machine learning approaches for subsurface characterization and decision-making.

## 3 Data Description

### 3.1 Content of the samples

**Fig. 1:**
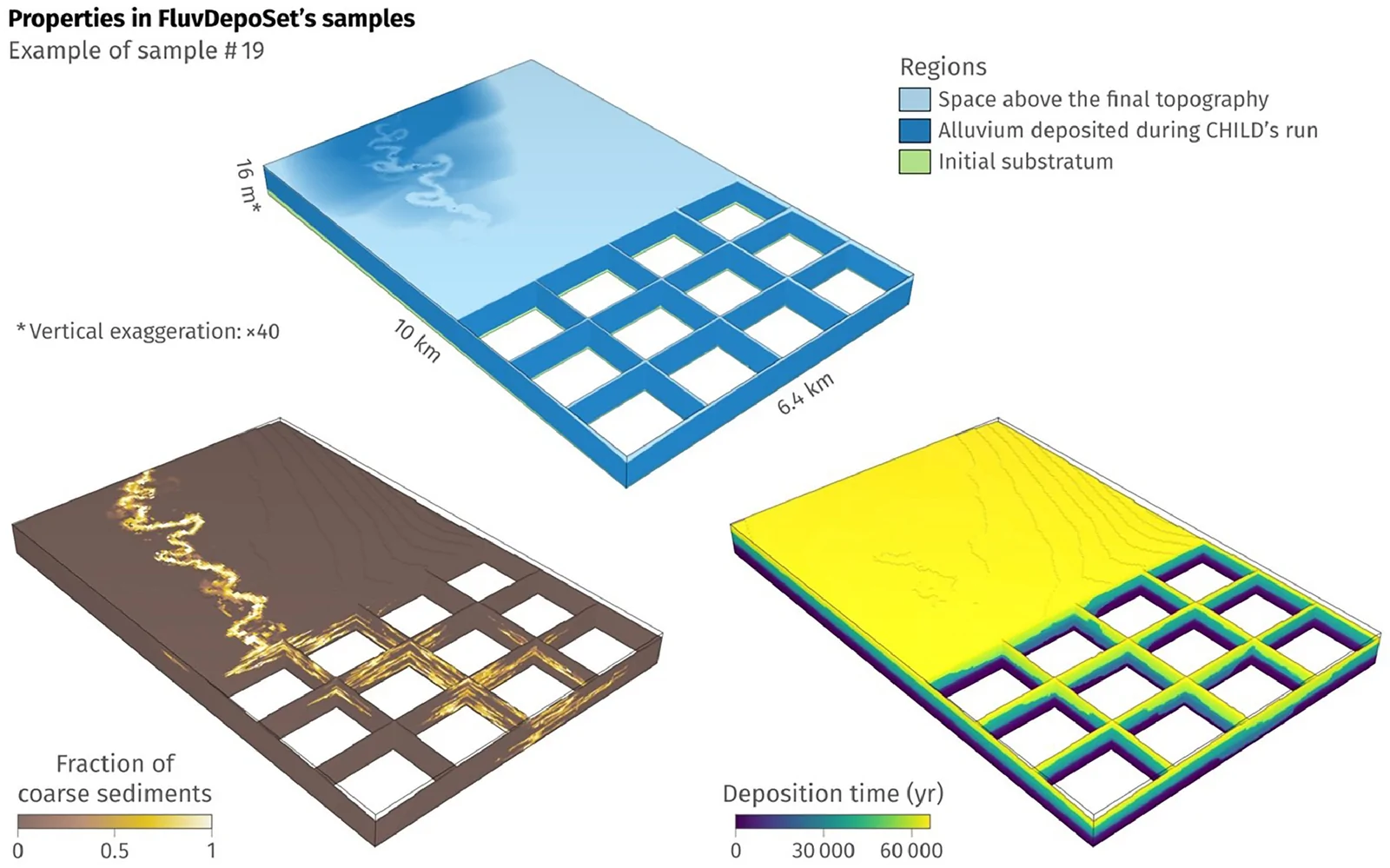
Example of sample from FluvDepoSet, with part of the 3D grid sliced to visualize the internal variability of each property. The corresponding input parameter values are highlighted in figure 3.

FluvDepoSet [6] includes 20,200 samples. Each sample consists of a 3D synthetic model of fluvial deposits in a regular structured grid, i.e., a 3D image (Fig. 1), with:

• 128 cells along x and 200 cells along y (horizontal dimensions), 32 cells along z (vertical dimension).
• A cell size of 50 m along both y and x, 0.5 m along z.
• An origin at 1000 m along both y and x, 0 m along z, based on where the grid is extracted from the original simulation.

Each model includes three properties:

1. The regions, which label the initial substratum, the simulated fluvial deposits, and the space above the final topography.
2. The fraction of coarse sediments, knowing that the simulations are based on two fractions, a fine and a coarse.
3. The deposition time of the sediments.

Those properties deviate from the datasets usually seen in geostatistics and machine learning for modeling fluvial deposits [e.g., 7,8,9], which only include facies, i.e., a discrete property representing clusters of rocks with similar characteristics. We decided to go beyond facies based on two considerations:

1. Facies result from an interpretation of subsurface data, so they are not a measurable quantity of direct value for subsurface applications. Properties based on grain size, such as the fraction of coarse sediments, are closer to real measurements, capture heterogeneities in more detail, and have a direct, physical link to other properties of interest, such as porosity and permeability.
2. On top of generating models consistent with geological principles, process-based models can give us extra information to support our interpretations of the local geology. The deposition time is an example of such information, because it is easier to deduce aggradation and incision phases – the history of the river evolution – from it than from the fraction of coarse sediments. Due to the high computational cost of process-based models, recent developments in generative machine learning are particularly attractive to use such properties in practice.

Nevertheless, it remains possible to generate facies based on thresholding the fraction of coarse sediments for instance.

### 3.2 Organization of the dataset

Each sample is stored in an independent HDF5 file that contains a single dataset under the key *model*. This dataset contains a 3D synthetic model of fluvial deposits as described in the previous subsection, so an array of shape (3, 32, 200, 128). The last three dimensions are the spatial dimensions z, y, and x, while the first dimension contains the three properties in the following order: (1) regions; (2) fraction of coarse sediments; (3) deposition time.

Each of those HDF5 files contains two levels of metadata:

• Three attributes at the file level that are the same for all samples:
  ◦ *dims*: the order of the spatial dimensions in the dataset and the other attributes, which is always (z, y, x).
  ◦ *extent*: the coordinates of the first and last grid node along each spatial dimension.
  ◦ *spacing*: the cell size along each spatial dimension.

Those attributes contain the minimum information missing from the dataset to use the sample, i.e., the grid dimensions.

• Seven attributes at the dataset level that vary for each sample, i.e., the values of the seven parameters that vary when generating a sample: BANKERO, FP_INLET_ELEVATION, FP_LAMBDA, FP_MU, GRAINDIAM1, GRAINDIAM2, and ST_PMEAN (see the next section for more detail about each parameter).

All those elements can be accessed in Python using the package *h5py* like so:

import h5py

with h5py.File('./sample_19.h5′) as file: sample = file['model'][:] parameters = dict(file['model'].attrs) dims = file.attrs['dims'] extent = file.attrs['extent'] spacing = file.attrs['spacing']

## 4 Experimental Design, Materials and Methods

### 4.1 Setting

**Fig. 2:**
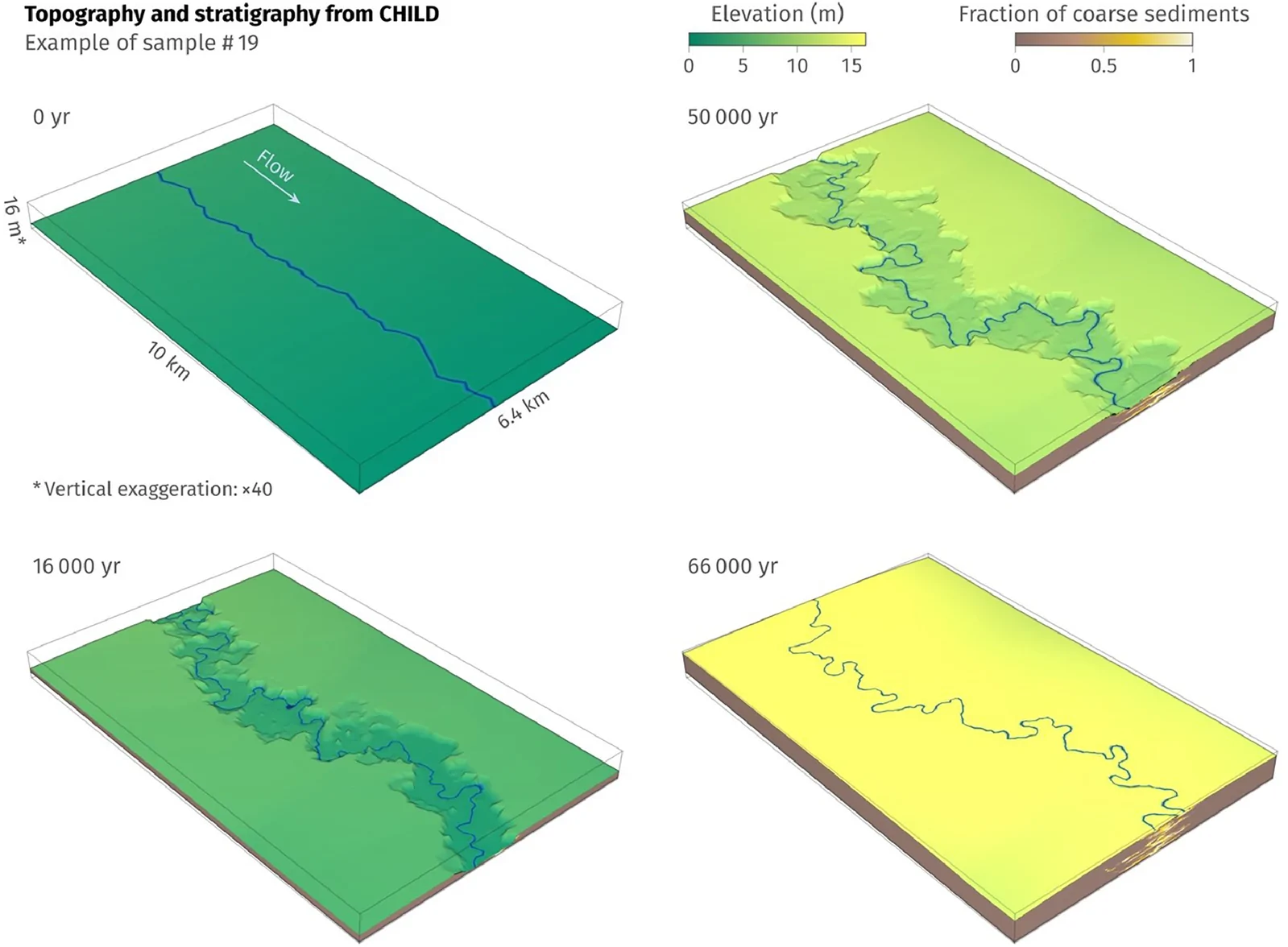
Generation of a sample using CHILD: a meandering river migrates while incising or aggrading; coarse sediments are deposited along the river, while fine sediments are deposited in the floodplain.

Models of fluvial deposits for machine learning or sensitivity analyses are often based on object-based approaches [e.g., 8,10], which randomly generate geological objects such as river channels in a domain of interest. While those approaches are flexible and relatively inexpensive in an unconditional context, they miss that geological objects do not develop randomly. Rivers for instance are continuously evolving: they migrate laterally, and aggrade or incise vertically. Thus, the deposits resulting from those processes follow specific patterns, and mimicking those processes is the most straightforward way to generate plausible distributions of fluvial deposits. As such, FluvDepoSet’s samples were generated by a landscape evolution model (Fig. 2), the Channel-Hillslope Integrated Landscape Development Model or CHILD for short [1,11], to obtain models consistent with geological principles.

CHILD uses a triangular irregular network (TIN) of nodes to represent the landscape surface. This irregular mesh prevents some biases observed on regular grids and allows horizontal displacements such as lateral migration. All samples are simulated from the same initial topography and boundary conditions. This initial topography is a 12 by 8.4 km flat domain with some small random noise and a slope of 3 × 10^−4^ m/m. A river inlet stands in the middle of the upper side of the domain, at an elevation of 3.6 m, while an outlet stands in the middle of the opposite side. Flow coming out of the inlet corresponds to a fixed drainage area of 2000 km^2^ and varies depending on rainfall.

To limit complexity and interpolation biases during simulation, fluvial deposits are recorded in a 3D curvilinear grid whose horizontal coordinates are fixed and regular [12]. This stratigraphic grid is only defined over a 10 by 6.4 km sub-domain to avoid border effects in the samples. It includes the fraction of coarse sediments and the deposition time, which are then interpolated into the final regular structured grid described in the previous section. This standardizes the format of all the samples and make potential subsequent operations – subsurface flow and transport simulation or machine learning training for instance – and comparisons easier. The interpolation consists in assigning the values of a layer from the curvilinear grid to all the cells of the regular grid whose centers are located inside that layer.

### 4.2 Data-generating process

Simulating physical processes over geological timescales can quickly become intractable, so CHILD uses empirical and simplified physical laws to reproduce efficiently the wide range of processes driving landscape evolution. Here, we focus more specifically on the four main processes influencing erosion and deposition from rivers: stochastic rainfall, lateral migration or meandering, overbank deposition, and aggradation and incision.

Rainfall is represented as a stochastic process based on the Poisson pulse rainfall model [13], which simulates a series of discrete storm events of constant rainfall intensity separated by inter-storm events without rainfall. Storm duration, storm rainfall intensity, and inter-storm duration follow exponential probability functions. Those probability functions are parameterized by a mean value, which can vary in time to introduce larger climatic trends. Water from rainfall all over the domain and from the river inlet gives rise to runoff, i.e., water flowing over the ground surface. This water is routed downslope using the steepest-descent method.

Lateral migration comes from erosion along the outer bank of a meander and deposition along its inner bank, forming point bars. Here, this process follows the topographic steering mechanism, in which variations in river-bed topography due to point bars steer the flow toward the outer bank, thus modifying the bank shear stress and driving erosion [14]:

(1) ζ=Eτn

with

$$\begin{array}{ccc} & & \zeta \;\text{bank}\;\text{migration}\;\text{rate}\;[L/T];\\ & & E\;\text{bank}\;\text{erodibility}\;[L/T\;\text{per}\;\text{unit}\;\text{stress}];\\ & & \zeta \;\text{bank}\;\text{shear}\;\text{stress}\;[F/L^{2}];\\ & & n\;\text{lateral}\;\text{unit}\;\text{vector}\;[\text{dimensionless}]. \end{array}$$

The bank shear stress depends on river bed sediments, river discharge, and river channel geometry [ for more details see 14]. Deposition along the inner bank remains implicit by assuming that the sediment supply is sufficient to preserve the channel width, a common approach to limit computational cost. The simulation mesh is adaptive to better handle erosion and deposition, so that eroded nodes along the outer bank are removed and new nodes are added along the inner bank after point bar deposition. These deposits are made of a single grain size corresponding to the coarse fraction of sediments. While this model greatly simplifies meandering processes, it has been shown to develop realistic meandering patterns [14], which is ultimately what matters here.

Overbank deposition deals with the second fraction of sediments deposited by a meandering river in CHILD: the fine fraction. While the coarse fraction is deposited in point bars along the river, the fine fraction is deposited in the floodplain by flood events. Here, this process follows the observation that the sedimentation rate in floodplains decreases exponentially with the distance from the river channel and is modeled as a diffusion process [1]:

(2) $$h_{sed}=\mu \left(z_{w}-z_{fp}\right)\exp\left(-\frac{d_{fp}}{\lambda }\right)$$

with

**Table:** 

$h_{sed}\;\;$ vertical deposition rate of fine sediments at a floodplain node [L/T];
μ overbank deposition rate constant [1/T];
$z_{w}\;\;$ elevation of floodwater at the nearest channel node [L];
$z_{fp}\;$ elevation of the floodplain location [L];
$d_{fp}\;$ distance between the floodplain location and the nearest channel node [L];
λ overbank distance decay constant [L].

The elevation of floodwater depends on discharge following Leopold & Maddock’s power-law hydraulic-geometry equations [15]:

(3) $$\begin{array}{ccc} z_{w} & = & z_{ch}+H_{w}\\ H_{w} & = & H_{b}{\left(\frac{Q}{Q_{b}}\right)}^{\delta_{s}}\\ H_{b} & = & k_{h}Q_{b}^{\delta_{b}} \end{array}$$

with

**Table:** 

$z_{ch}\;$ bed elevation at the nearest channel node [L];
$H_{w}\;$ water depth at the nearest channel node [L];
$H_{b}\;\;$ bankfull channel depth [L];
Q discharge at the nearest channel node $\left[L^{3}/T\right]$;
$Q_{b}\;\;$ bankfull discharge $\left[L^{3}/T\right]$;
$\delta_{s}\;\;$ at-a-station depth scaling exponent [dimensionless];
$\delta_{b}\;\;$ downstream depth scaling exponent [dimensionless];
$k_{h}\;\;$ downstream depth coefficient $\left[L\cdot L^{-3}\cdot T^{-1}\right]$.

Runoff, river channel path, river geometry, and lateral migration are updated after each storm event, while overbank deposition only occurs when storm intensity is high enough that $H_{w}>H_{b}$.

Aggradation corresponds to an increase in elevation due to the vertical accumulation of sediments, incision to a decrease in elevation due to the vertical erosion of sediments. In CHILD's meandering mode, they are implemented as a boundary condition: the elevation of the river inlet can vary up and down through time to depict externally imposed aggradation and incision phases (Fig. 2). This geometrical approach provides a more efficient solution than including the processes driving aggradation and incision, such as tectonics and climate [16].

### 4.3 Input parameter values

**Fig. 3:**
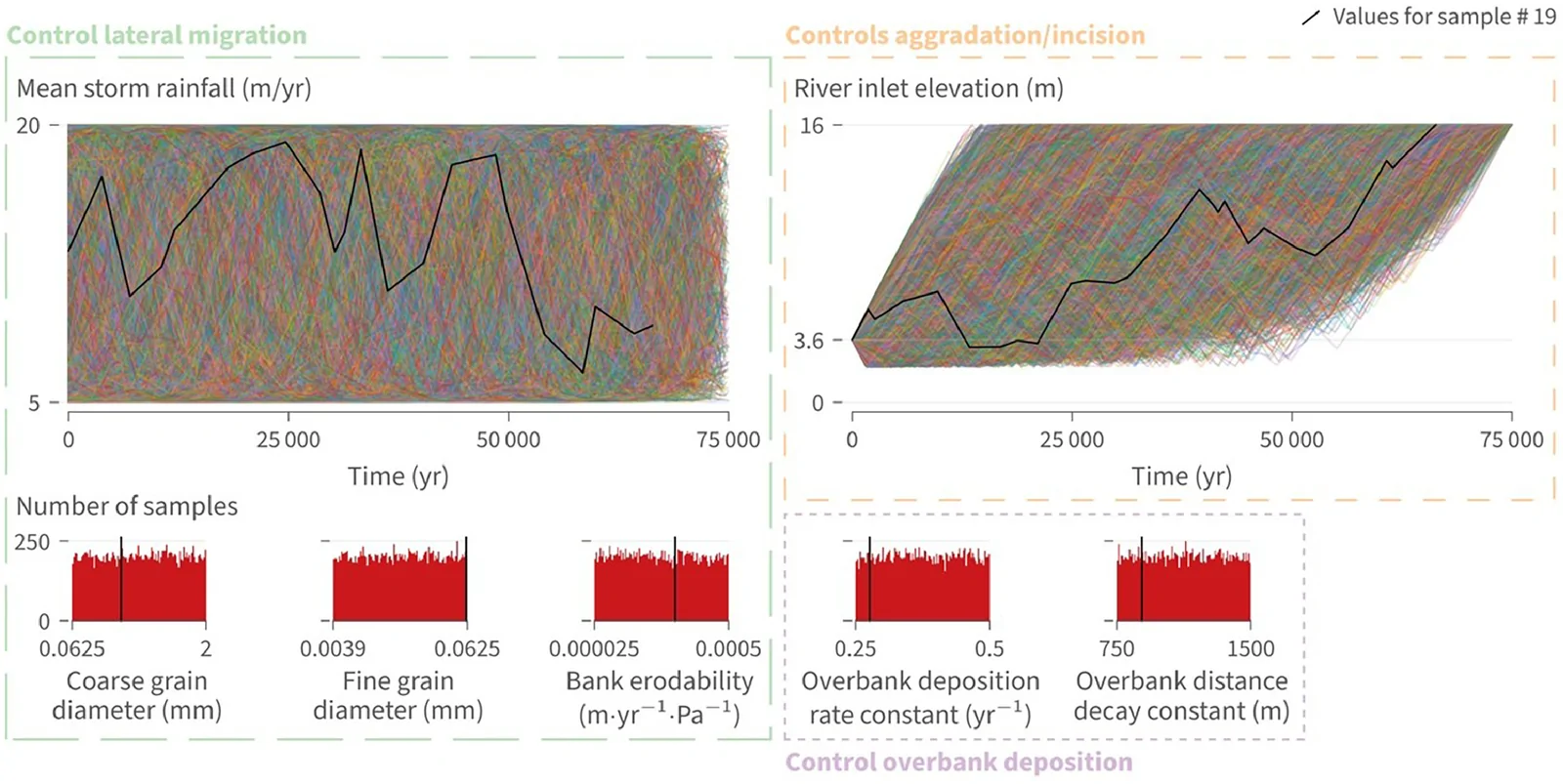
Values of the input parameters of CHILD for the 20,200 samples of FluvDepoSet.

Preserving the robustness of sensitivity analyses requires to drastically increase the number of samples when the number of parameters to analyze increases. Considering that generating a sample can take tens of hours, we had to focus on a handful of parameters (Fig. 3) that govern the processes described in the previous subsection. All the other parameters are kept constant (see table A1 that lists the values of all the parameters).

All the inputs that vary between samples come from uniform distributions that aim at exploring a parameter space as large as possible while enabling river meandering:

• BANKERO: Bank erodibility used in equation 1. This parameter amalgamates several concepts beyond sediment erodibility along the banks, such as the presence and type of vegetation, making it difficult to estimate in practice. Its values are based on previous studies using CHILD’s meandering mode [e.g., 12,16] and our own experiments.
• FP_INLET_ELEVATION: River inlet elevation through time used to control aggradation and incision. We randomly draw time series based on periods ranging from 500 to 5000 years and aggradation rates from −1 to 1 m/kyr, a negative value meaning incision, following the model of Clevis et al. [16]. We use a rejection sampler to select only the time series in which the inlet reaches 16 m without going below 2 m in <75,000 years to limit the computational burden. This leads to an average thickness for a channel belt in a meandering setting. All the samples have the same final inlet elevation but different simulation times.
• FP_LAMBDA: Overbank distance decay constant used in equation 2. Its values are based on previous studies using CHILD’s meandering mode [e.g., 12,16] and our own experiments.
• FP_MU: Overbank deposition rate constant used in equation 2. Its values are based on previous studies using CHILD's meandering mode [e.g., 12,16] and our own experiments.
• GRAINDIAM1: Coarse grain diameter used in the bank shear stress of equation 1 and for deposition in point bars. Its values correspond to sand on Wentworth’s scale (0.0625 to 2 mm) [17].
• GRAINDIAM2: Fine grain diameter used in the bank shear stress of equation 1 and for deposition in the floodplain. Its values correspond to silt on Wentworth’s scale (0.0039 to 0.0625 mm) [17].
• ST_PMEAN: Mean storm rainfall through time used to control runoff and discharge. Rainfall parameters come from estimates of the parameters for the Poisson pulse rainfall model over the continental United States [18], which cover a large variety of climates and are often used in studies using CHILD [e.g., 13,19]. While we keep storm and inter-storm duration constant, the mean storm rainfall randomly varies between 5 and 20 m/yr over periods from 500 to 5000 years with a correlation of 0.75 between two successive values to limit abrupt variations.

## Limitations

While FluvDepoSet captures a large variety of spatial distribution of fluvial deposits, it is limited to a specific setting: deposits with two sediment fractions from a single-threaded, sand-bedded meandering river at the scale of a single channel belt. In reality, fluvial deposits contain more sediment classes than just a fine and a coarse fraction and exhibit different heterogeneity distributions when formed by braided or anastomosing rivers. Additionally, avulsions can create multiple channel belts that further complicate the heterogeneity of those deposits. This restricts the use of FluvDepoSet in real case studies.

CHILD can still generate avulsions because it recomputes the river channel path at each iteration rather than using a fixed path throughout the simulation. While we set the parameters so that avulsions are unlikely, they can still occur in some samples. In some rare cases, we have observed that CHILD flags too many nodes as river channel nodes after an avulsion, resulting in the deposition of large sheets of coarse sediments. These deposits do not match our setting, but we decided to keep these samples. They can be useful to develop and test algorithms to pre-process such large datasets.

## Ethics Statement

Our study follows the ethical requirements of Data in Brief and we confirm that it does not involve human subjects, animal experiments, or any data collected from social media platforms.

## CRediT Author Statement

**Guillaume Rongier:** Conceptualization, Methodology, Software, Writing - Original Draft, Writing - Review & Editing. **Luk Peeters:** Conceptualization, Writing - Review & Editing, Supervision.

## Figures and Tables

**Table A1:** CHILD parameter values used to generate the samples. The parameters in red vary for each sample. See [20] for more detail about each parameter. ^t^: time-series parameter; U(min, max): uniform distribution and bounds.

Parameter	Value	Parameter	Value
RUNTIME	Final time of FP_INLET_ELEVATION	HYDR_ROUGH_EXP_STN	−0.21
OPINTRVL	Final time of FP_INLET_ELEVATION	BANK_ROUGH_COEFF	75
SEED	12	BANK_ROUGH_EXP	0
OPTREADINPUT	10	BANKFULLEVENT	12 m·yr^−1^
X_GRID_SIZE	8400 m	OPTMEANDER	1
Y_GRID_SIZE	12,000 m	CRITICAL_AREA	2 × 10^9^ m^2^
OPT_PT_PLACE	1	OPT_VAR_SIZE	1
GRID_SPACING	200 m	BANKERO	U(0.000025, 0.0005) m·yr^−1^·Pa^−1^
INPUTTIME	n/a	BNKHTDEP	0
OPTMESHADAPTAREA	0	DEF_CHAN_DISCR	1 m
OPTREADLAYER	0	FRAC_WID_MOVE	0.1 channel width
OPTMESHADAPTDZ	0	FRAC_WID_ADD	0.7 channel width
TYP_BOUND	4	OPTNOFLUVIAL	1
NUMBER_OUTLETS	1	DETACHMENT_LAW	1
OUTLET_X_COORD	4200 m	KB	0.0005 [see 20 for units]
OUTLET_Y_COORD	0.01 m	KR	0.0005 [see 20 for units]
MEAN_ELEV	0 m	KT	1000 [see 20 for units]
RAND_ELEV	0.01 m	MB	0.66667
SLOPED_SURF	1	NB	0.66667
UPPER_BOUND_Z	3.6 m	PB	1.5
OPTINLET	1	TAUCB	30 [see 20 for units]
INDRAREA	2 × 10^9^ m^2^	TAUCR	30 [see 20 for units]
INSEDLOAD1	0.2	BETA	1
INSEDLOAD2	0.8	OPTDETACHLIM	0
INLET_X	4200 m	TRANSPORT_LAW	1
INLET_Y	11,999.99 m	KF	617 [see 20 for units]
INLET_OPTCALCSEDFEED	1	MF	0.66667
INLET_SLOPE	2 × 10^−4^ m·m^−1^	NF	0.66667
ROCKDENSITYINIT	2270 kg·m^−3^	PF	1.5
SOILBULKDENSITY	740 kg·m^−3^	OPTNODIFFUSION	0
NUMGRNSIZE	2	KD	10^−3^ m^2^·yr^−1^
GRAINDIAM1	U(0.0000625, 0.002) m	OPTDIFFDEP	0
GRAINDIAM2	U(0.0000039, 0.0000625) m	DIFFUSIONTHRESHOLD	0 m^2^
REGPROPORTION1	0	OPT_NONLINEAR_DIFFUSION	0
REGPROPORTION2	1	OPT_DEPTH_DEPENDENT_DIFFUSION	1
BRPROPORTION1	0	DIFFDEPTHSCALE	1
BRPROPORTION2	0	OPTFLOODPLAIN	1
BEDROCKDEPTH	10^8^ m	FP_DRAREAMIN	2 × 10^9^ m^2^
REGINIT	10^8^ m	FP_BANKFULLEVENT	12 m·yr^−1^
MAXREGDEPTH	1 m	FP_MU	U(0.25, 0.5) yr^−1^
OPT_READ_LAYFILE	0	FP_LAMBDA	U(750, 1500) m
OPT_READ_ETCHFILE	0	FP_OPTCONTROLCHAN	1
OPT_SET_ERODY_FROM_FILE	0	FP_VALDROP	2 m
OPTLAYEROUTPUT	0	FP_INLET_ELEVATION^t^	
OPTINTERPLAYER	0	Initial value	3.6 m
OPTVAR	1	Final value	16 m
ST_PMEAN^t^		Minimum value	2 m
Value	U(5, 20) m·yr^−1^	Time step	U(500, 5000) yr
Autocorrelation	0.75	Maximum simulation time	75,000 yr
Time step	U(500, 5000) yr	Aggradation rate	U(−0.001, 0.001) yr
Simulation time	From FP_INLET_ELEVATION	Mode	Interpolate
Mode	Interpolate	OPTSTRATGRID	1
ST_STDUR	0.09 yr	XCORNER	975 m
ST_ISTDUR	1 yr	YCORNER	975 m
ST_OPTSINVAR	0	GRIDDX	50 m
OPTSINVARINFILT	0	GR_WIDTH	6500 m
OPTNOUPLIFT	1	GR_LENGTH	10,100 m
FLOWGEN	0	SG_MAXREGDEPTH	0.5 m
INFILTRATION	0 m·yr^−1^	CHEM_WEATHERING_LAW	0
OPTSINVARINFILT	0	PRODUCTION_LAW	0
PERIOD_INFILT	0 yr	OPT_LANDSLIDES	0
MAXICMEAN	0 m·yr^−1^	OPT_3D_LANDSLIDES	0
LAKEFILL	1	OPTLOESSDEP	0
CHAN_GEOM_MODEL	1	OPTVEG	0
HYDR_WID_COEFF_DS	3	OPTFOREST	0
HYDR_WID_EXP_DS	0.5	OPTFIRE	0
HYDR_WID_EXP_STN	0.26	OPTTSOUTPUT	0
HYDR_DEP_COEFF_DS	0.028	SURFER	0
HYDR_DEP_EXP_DS	0.4	OPT_TRACK_WATER_SED_TIMESERIES	0
HYDR_DEP_EXP_STN	0.4	OPT_FREEZE_ELEVATIONS	0
HYDR_ROUGH_COEFF_DS	0.03	OPTSTREAMLINEBNDY	0
HYDR_ROUGH_EXP_DS	−0.01		

## Data Availability

CSIRO Data Access PortalFluvDepoSet (Original data)
